# Assessment of surface roughness and blood rheology on local coronary haemodynamics: a multi-scale computational fluid dynamics study

**DOI:** 10.1098/rsif.2020.0327

**Published:** 2020-08-12

**Authors:** David G. Owen, Torsten Schenkel, Duncan E. T. Shepherd, Daniel M. Espino

**Affiliations:** 1 Department of Mechanical Engineering, University of Birmingham, UK; 2 Department of Engineering and Mathematics, Materials and Engineering Research Institute MERI, Sheffield Hallam University, Sheffield, UK

**Keywords:** computational fluid dynamics, coronary, multiphase, red blood cell migration, rheology, roughness

## Abstract

The surface roughness of the coronary artery is associated with the onset of atherosclerosis. The study applies, for the first time, the micro-scale variation of the artery surface to a 3D coronary model, investigating the impact on haemodynamic parameters which are indicators for atherosclerosis. The surface roughness of porcine coronary arteries have been detailed based on optical microscopy and implemented into a cylindrical section of coronary artery. Several approaches to rheology are compared to determine the benefits/limitations of both single and multiphase models for multi-scale geometry. Haemodynamic parameters averaged over the rough/smooth sections are similar; however, the rough surface experiences a much wider range, with maximum wall shear stress greater than 6 Pa compared to the approximately 3 Pa on the smooth segment. This suggests the smooth-walled assumption may neglect important near-wall haemodynamics. While rheological models lack sufficient definition to truly encompass the micro-scale effects occurring over the rough surface, single-phase models (Newtonian and non-Newtonian) provide numerically stable and comparable results to other coronary simulations. Multiphase models allow for phase interactions between plasma and red blood cells which is more suited to such multi-scale models. These models require additional physical laws to govern advection/aggregation of particulates in the near-wall region.

## 1. Introduction

The progression and impact of cardiovascular disease (CVD) is often directly related to the health of the coronary arteries [1]. CVD is the leading cause of death worldwide [2] accounting for 31% of global deaths in 2016, as well as being detrimental to other conditions [3]. Recent advances in computational fluid dynamics (CFD) have allowed for detailed study into coronary haemodynamics [4,5], in particular, the relationship between atherosclerosis and flow parameters such as wall shear stress [6–9] (WSS). A detailed overview on the use of patient-specific models by Taylor and Figueroa [10] highlighted many clinical applications [11–15], with the most relevant to this study being predictions in the progression of atherosclerosis [16,17], arterial fibrin clots [18] and thrombus formation [19], as well the effect of arterial stenosis on blood flow [20–22]. The lumen wall is assumed smooth for all coronary models to date. Studies into the roughness of the coronary lumen have shown that its texture plays a role in the early formation of atherosclerosis [23–25], with an increase in roughness resulting from endothelial damage [26]. Accurate estimation of *in vivo* lumen roughness using ultrasound may be useful clinically for diagnostic purposes, however, this is challenging, with limitations on resolution [26]. Furthermore, other imaging methods and geometry processing techniques under current clinical use are unable to capture the surface roughness of coronary arteries. However, the surface roughness (Ra) of porcine coronary arteries have recently been characterized *ex vivo* using optical microscopy [27–29], atomic force microscopy and scanning electron microscopy [30].

Blood is a heterogeneous, thixotropic fluid which presents challenges to its accurate simulation using CFD. It is comprised of red blood cells (RBCs) and other cells (e.g. monocytes, leucocytes, etc.) suspended within a plasma continuum [31]. A comprehensive review of computational approaches to blood modelling is provided by Bessonov *et al.* [32]. One approach taken to simulate blood is to model it using a single-phase rheological model. Single-phase models treat blood as a homogeneous fluid, with either a constant (Newtonian) viscosity or a shear-dependent viscosity (non-Newtonian). Non-Newtonian blood viscosity models enable the shear-thinning behaviour of blood to be modelled; however, blood is treated as a single constituent fluid. This neglects the contribution of the cellular phases (RBCs, platelets, white blood cells, etc.) suspended within the plasma, which at lower shear rates often aggregate causing rouleaux formation [33] and greatly increase the viscosity of the mixture [34]. Multiphase models, instead, can be used to simulate blood as dilute suspension of RBCs within a plasma continuum. Thus, the flow of RBCs can be distinguished from that of the surrounding plasma. These multiphase models often use an Eulerian–Eulerian approach which has previously been used in other simulations [35–40] to investigate cardiovascular pathologies, evolving theory developed by Gidaspow [31] with an assessment of drag/lift/mass modelling of RBCs for cardiovascular modelling by Yilmaz *et al*. [34]. An advantage of the multiphase models is their ability to capture local variations in haematocrit (RBC concentration) arising from fluid dynamics and can then apply this to the viscosity of the blood, compared to the assumed uniform distribution of the single-phase models. No current single or multiphase CFD models of blood flow through coronary arteries simulate surface roughness. Therefore, it is unknown how predictions from these models vary with surface topology.

The current study aims to investigate, for the first time, how a realistically rough walled segment of the coronary artery impacts on the well-established haemodynamic parameters used to assess coronary health and how roughness might impact the onset and progression of atherosclerosis. A comparison of common approaches to blood rheology has been performed to assess their capability of modelling flow features at a micro-scale. Briefly, three approaches to modelling blood rheology are compared: a Newtonian blood model, non-Newtonian single-phase models (Carreau, Carreau–Yasuda, and generalized power law), and multiphase models (Quemada–Das and MKM5). The models are compared using transient simulations of blood flow through a simplified macro-scale coronary artery, with comparisons focusing on how flow over a smooth segment of the artery differs to flow over a segment which includes micro-scale roughness.

## 2. Methods

### 2.1. Geometry

#### 2.1.1. Artery segmentation

**Figure 1.**
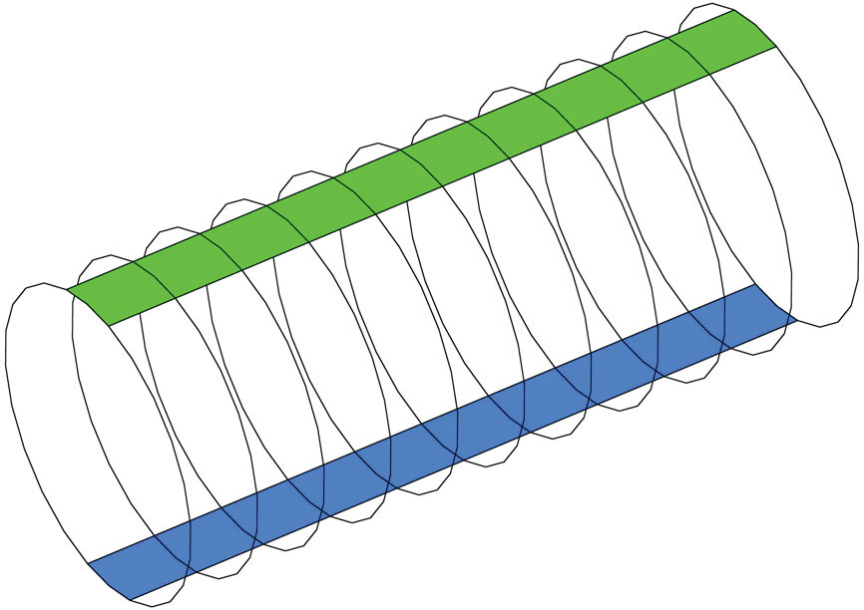
Partially rough coronary artery with identical rough/smooth sections defined opposite.

This model considers an idealized, short cylindrical section of the left anterior descending (LAD) coronary artery with a constant diameter of 3.5 mm chosen based upon *in vivo* data [41–43]. The artery wall is predominantly smooth, with a rough-surfaced segment of arc length 0.8 mm (26.2°) along the length of the artery. It is constructed from 10, 0.8 mm long segments giving a total length of 8 mm. Ten segments were sufficient for the roughness to impact on the local haemodynamics without being overly computationally intensive. For best comparisons between the rough and smooth walls within the same model, an identical smooth segment is computationally defined directly opposite the rough wall (figure 1), with all results being evaluated over a 4 mm length starting 2.4 mm from the inlet.

#### 2.1.2. Rough surface generation

**Figure 2.**
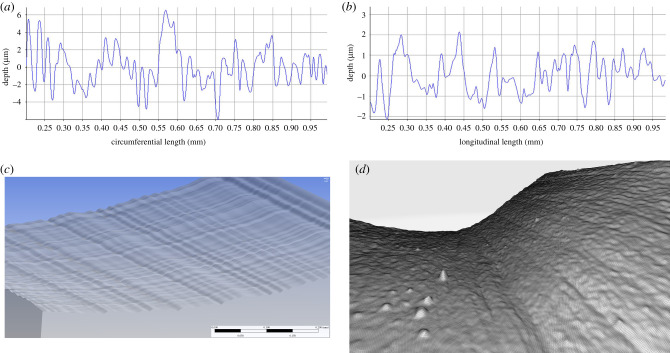
(*a*,*b*) Circumferential and longitudinal height profiles respectively. (*c*) Rough surface on 0.8 mm artery segment. (*d*) Representative surface roughness taken from optical microscopy. (Spikes are imaging artefacts, their causes are explained by Burton *et al*. [30].)

The surface roughness of the porcine coronary LAD artery in the circumferential and longitudinal directions have been reported as *Ra_C_* = 1.04 ± 0.45 μm and *Ra_L_* = 0.89 ± 0.27 μm, respectively, with no variation along the length of the artery [28]. To generate the rough surface, representative profiles of surface height (figure 2*a*,*b*) in each direction were segmented into 16 × 0.05 mm sections and fitted to cubic B-splines. The circumferential profile was applied along a 0.8 mm section of the arterial circumference, with the longitudinal profile running perpendicularly (along the length of the artery). By extruding these two profiles along each other, an idealized three-dimensional (3D) rough surface (figure 2*c*) can be created and then repeated to form the rough section of the artery. A comparison between the surface generated using the height profiles from the literature [27] and a representative sample of the porcine artery surface from optical microscopy data is shown in figure 2*c*,*d*.

### 2.2. Mesh

**Figure 3.**
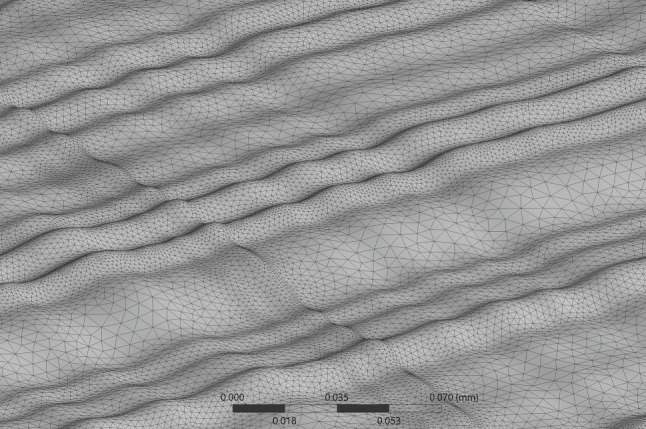
Variation of mesh size on a portion of the rough segment.

A detailed mesh is required to capture the smooth variation in surface texture. This was achieved using a combination of both tetrahedral and prism elements varying in size due to curvature, with elements having a minimum edge length of 0.2 µm and 18 inflation layers around the lumen surface. This was chosen so that the near-wall haemodynamics on the rough artery were computed over several elements. To ensure a mesh-independent solution, simulations were performed on a shortened model at six equally spaced increasing levels of mesh refinement. The area-averaged WSS over the rough surface was calculated for each mesh refinement level, until the percentage difference between each refinement was below 0.5%. This resulted in a 65 million element mesh (figure 3) with an average orthogonal quality and skewness of 0.77 and 0.22, respectively [44].

### 2.3. Rheological models

**Figure 4.**
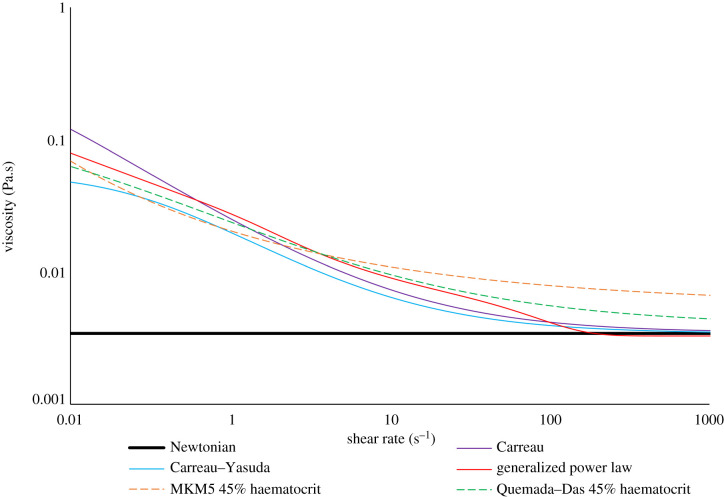
Variation in model viscosity for varying shear rate.

Blood exhibits both Newtonian and non-Newtonian properties which can be described by fitting experimental viscometer data [45–48] to constituent viscous definitions which can be functions of the volume fraction of the RBCs (haematocrit) or, more typically, just the shear rate of the fluid (equation (2.1)). This study will examine six models, with the effective model viscosity under varying shear given in figure 4 and the accompanying model definitions in table 1.

2.1 $$\dot{\gamma }=\sqrt{2D_{ij}\cdot D_{ij}},$$

where $\dot{\gamma }\;$ is the shear rate of the fluid, *D* is the strain rate tensor with *i*, *j* = 1, 2, 3 as the inner products.

**Table 1.** Rheological model definitions and parameters.

model	viscosity definition (Pa.s)	parameters
Newtonian [49]	μ=0.00345	—
Carreau [50]	$\mu =\mu_{\infty }+(\mu_{\infty }+\mu_{0}){\left[1+{\left(\lambda \dot{\gamma }\right)}^{2}\right]}^{(n-1)/2}$	$\begin{array}{rl} & \mu_{\infty }=0.00345,\;n=0.25\\ & \;\mu_{0}=0.025,\;\lambda =25\; \end{array}$
Carreau–Yasuda [51]	$\mu =\mu_{\infty }+(\mu_{\infty }+\mu_{0}){\left[1+{\left(\lambda \dot{\gamma }\right)}^{p}\right]}^{(n-1)/p}$	$\begin{array}{rl} & \mu_{\infty }=0.00345,\;n=0.22\\ & \;\mu_{0}=0.056,\;p=1.25\\ & \;\;\lambda =1.902\; \end{array}$
generalized power law [52]	$\begin{array}{rl} \mu =\; & k(\dot{\gamma })|\dot{\gamma }{|}^{n(\dot{\gamma })-1}\\ k(\dot{\gamma }\;)= & \mu_{\infty }+\Delta \;\mu \cdot \exp\left[-\left(1+\frac{\left|\dot{\gamma }\right|}{a}\right)\exp\left(\frac{-b}{\dot{\gamma }}\right)\right]\\ n(\dot{\gamma }\;)=\; & n_{\infty }-\Delta n\cdot \text{exp}\left[-\left(1+\frac{\left|\dot{\gamma }\right|}{c}\right)\exp\left(\frac{-d}{\dot{\gamma }}\right)\right] \end{array}$	$\begin{array}{rl} \mu_{\infty } & =0.0035,\Delta \mu =0.25\\ n_{\infty } & =1,\;dn=0.45\\ a & =c=50,b=3,d=4 \end{array}$
Quemada–Das [53]	$\mu =\mu_{p}{\left[1-\frac{k\emptyset_{rbc}}{2}\right]}^{-2},$ $k=\frac{k_{0}+k_{\infty }\sqrt{\dot{\gamma }/\gamma_{c}}}{1+\sqrt{\dot{\gamma }/\gamma_{c}}},$ $k_{0}=a_{0}+\frac{2}{a_{1}+\emptyset_{rbc}},$ $k_{\infty }={\text{e}}^{b_{0}+b_{1}\emptyset_{rbc}+b_{2}\emptyset_{rbc}^{2}+b_{3}\emptyset_{rbc}^{3}},$ $\gamma_{c}={\text{e}}^{c_{0}+c_{1}\emptyset_{rbc}+c_{2}\emptyset_{rbc}^{2}+c_{3}\emptyset_{rbc}^{3}}$	$\mu_{p}=0.00123$ $a_{0}=0.06106$ $a_{1}=0.04777$ $b_{0}=1.80096$ $b_{1}=-3.66602$ $b_{2}=2.57412$ $b_{3}=0.02346$ $c_{0}=-7.01332$ $c_{1}=34.38771$ $c_{2}=-39.80154$ $c_{3}=13.99167$ $Not\;valid\;for\;k\emptyset_{rbc}=2,\;yet\;this$ $occurs\;when\;0>\emptyset_{rbc}>1$
modified Krieger model 5 parameters [54] (MKM5)	$\mu =\mu_{p}{\left[1-\frac{\emptyset_{rbc}}{\emptyset_{rbc,crit}}\right]}^{-n}$ $n=\{\begin{array}{ccc} n_{\infty } & \text{if} & \emptyset_{rbc}<0.2\\ n_{\infty }+n_{st} & \text{if} & \emptyset_{rbc}>0.2 \end{array}$ $n_{\infty }=be^{-c\emptyset_{rbc}},\;n_{st}=\beta \gamma^{\;\prime -\nu }$ ${\gamma \;}^{\prime }=1+{\left(\;\lambda \dot{\gamma }\right)}^{\nu_{g}}$	$\mu_{p}=0.00123$ $\emptyset_{rbc,crit}=0.95$ b=8.78084 c=2.82354 β=16.27775 ν=0.14275 λ=1252.64407 $\nu_{g}=2$

Where *μ_p_* is the viscosity of plasma, $\emptyset_{rbc}\;$ is the haematocrit (phase volume fraction) and $\emptyset_{rbc,\mathrm{crit}}\;$ is the critical haematocrit for which the RBCs no longer behave as a fluid. The Newtonian model assumes a constant viscosity, both Carreau models use a simplistic asymptotic polynomial shear-thinning definition and the generalized power-law model combines the Casson [55] and Carreau models with a simple power law. The final two models (Quemada–Das and MKM5) are based upon multiphase mixture theory where RBC's viscosity varies with both local shear forces and haematocrit [56]. The MKM5 model parameters have been revised [57] based upon a curve fitting to the Brooks *et al.* [45] experimental data. As both multiphase models above describe the viscosity of whole blood, to determine the intrinsic viscosity of the RBC's themselves, equation (2.2) below is used;

2.2 $$\mu_{rbc}=\frac{\mu_{\mathrm{blood}}-(1-\emptyset_{rbc})\mu_{p}}{\emptyset_{rbc}},$$

where *μ*_blood_ is the definition of viscosity in the Quemada–Das and MKM5 models as shown in table 1.

## 3. Numerical methods

### 3.1. Governing equations

#### 3.1.1. Single-phase Navier–Stokes

The commercial finite volume (FV) solver Fluent (Ansys v. 19.2, Ansys Inc, PA, USA) was used to solve the Navier–Stokes equations governing the behaviour of a 3D incompressible fluid (equations (3.1) and (3.2)) for all of four the single-phase models (Newtonian, Carreau, Carreau–Yasuda and generalized power law).

3.1 $$\frac{\partial u_{i}}{\partial x_{i}}=0$$

and

3.2 $$\frac{\partial u_{i}}{\partial t}+u_{j}\frac{\partial u_{i}}{\partial x_{j}}=\frac{\partial }{\partial x_{j}}\left(\mu \frac{\partial u_{i}}{\partial x_{j}}\right)-\frac{1}{\rho }\frac{\partial p}{\partial x_{i}},$$

where *u_i_* is the velocity vector in the *i* direction, *p* is the fluid pressure, *ρ* is the density of the fluid, *x* is the coordinate in *i* or *j* direction and *μ* is the fluid viscosity and *t* is time. The viscous definitions for these models is given in table 1, with the density of blood [58–60] set as *ρ* = 1060 kg m^−3^.

#### 3.1.2. Multiphase rheological formulation

A Eulerian–Eulerian multiphase mixture model was implemented, which considers blood as a composition of a Newtonian continuum (plasma) with a non-Newtonian particulate suspension (RBCs) whose viscosity depends on local shear and haematocrit. With RBCs accounting for greater than 99% of the cellular volume fraction [61] other particulates are not considered here. The fundamental laws governing phase volume fraction, continuity of mass/momentum for primary/secondary phases *p*, *q* = *plasma*, *rbc* are given, respectively, in equations (3.3)–(3.5).

3.3 $$\overset{2}{\underset{n=1}{\sum }}\emptyset_{n}=1\;,$$

3.4 $$\frac{\partial }{\partial t}(\emptyset_{q}\rho_{q})+\nabla \cdot (\emptyset_{q}\rho_{q}{\vec{v}}_{q})=0$$

and

3.5 $$\begin{array}{rl} \frac{\partial }{\partial t}(\emptyset_{q}\rho_{q}v_{q})+\nabla \cdot (\emptyset_{q}\rho_{q}v_{q}v_{q}) & =-\emptyset_{q}\nabla \text{p}+\nabla \cdot {\bar{\bar{\tau }}}_{q}\\ & \;+\overset{2}{\underset{\;p,q=1}{\sum }}K_{\;pq}(v_{p}-v_{q})\\ & \;+F_{\mathrm{ext}}, \end{array}$$

where *ρ* is density, ***v*** is velocity, *p* is pressure (shared by all phases), $\;\bar{\bar{\tau }}$ is the stress–strain tensor, *K_pq_* is the interphase momentum exchange coefficient and ${\vec{F}}_{ext}\;$are the external forces. The viscous definitions in table 1 coupled with phase definitions for the density of plasma [45] and RBCs [62] being *ρ_p_* = 1003 kg m^−3^ and *ρ_q_* = 1096 kg m^−3^, respectively, leaves only definitions of ***F***_ext_ and *K_pq_* to be described. The main consideration is the exchange of momentum between the two phases which is closely coupled to the viscous drag experienced by the RBCs. This relationship can be derived from the particulate phases interfacial area [63], and drag force given by the Schiller–Naumann [64] model for spheres as

3.6 $$K_{\;pq}=\frac{3}{4}C_{D}\frac{\rho_{p}\emptyset_{q}(1-\emptyset_{q})|{\vec{v}}_{p}-{\vec{v}}_{q}|}{d_{q}}$$

and

3.7 $$C_{D}=\{\begin{array}{ccc} \frac{24(1+0.15Re^{0.687})}{Re} & \text{if} & Re\leq 1000\\ 0.44 & \text{if} & Re>1000 \end{array},$$

where *d_q_* is the diameter of the RBC [32,65] chosen to be 8 µm, *C_D_* is the drag coefficient for the RBC and *Re* is the relative Reynolds number which is defined as

3.8 $$Re=\frac{\rho_{p}|v_{p}-v_{q}|d_{q}}{\mu_{p}}.$$

In addition to this, a ‘virtual mass’ force is included which concerns the change in inertia of the plasma during relative RBC acceleration [66] which is defined as

3.9 $$F_{vm}=0.5\emptyset_{q}\rho_{p}\left(\frac{{\text{d}}_{p}v_{p}}{\text{d}t}-\frac{{\text{d}}_{q}v_{q}}{\text{d}t}\right),$$

where the term *d_p_*/d*t* represents the phase material time derivative of the form:

3.10 $$\frac{d_{p}(\emptyset )}{\text{d}t}=\frac{\partial (\emptyset )}{\text{d}t}+(v_{p}\cdot \nabla )\emptyset .$$

The lift force is not included in this model as it is not appropriate for models with such a high concentration of small particles (relative to the artery), and is more applicable to larger dispersed flows in which highly separable flow occurs [34].

### 3.2. Boundary conditions

#### 3.2.1. Inlet and outlet

The pulsatile variation of velocity over time, *V*(*t*), was applied to the inlet using a Fourier fitted series (*R*^2^ = 0.998) based upon velocity data for the LAD [67].

A simplified pulsatile model (equation (3.11)) has been used to describe the fully developed laminar flow occurring at the inlet as recommended by Chabi *et al.* for modelling micro-scale features [68] (stents).

**Figure 5.**
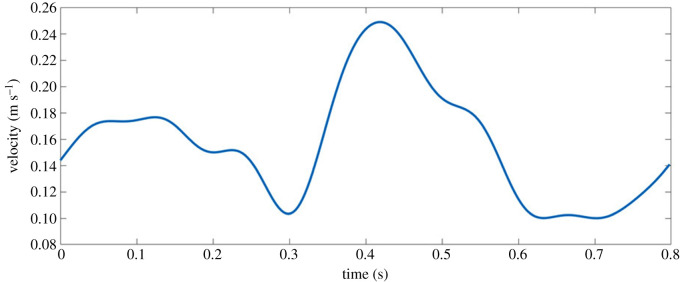
Variation of velocity for inlet to coronary artery.

3.11 $$u(r,t)=2V(t)\left[1-\left(\frac{r^{2}}{R^{2}}\right)\right],$$

where *V*(*t*) is the variation of velocity shown in figure 5, *r* is the radial position and *R* is the maximum radius of the artery (adjusted for the increase in diameter due to the roughness). Due to the short length of the artery, a transient outlet boundary condition was not employed, instead, the outlet had a constant 75 mmHg reference pressure applied based upon *in vivo* measurements [69] which have been implemented in similar coronary models [35,36,70].

#### 3.2.2. Multiphase haematocrit

An initial uniform haematocrit distribution of 45% is assumed at the inlet based upon the healthy adult range [71] of 0.47 ± 0.07 with other similar studies also using this uniform distribution [35–37,72]. This uniform distribution is assumed due to a lack of *in vivo* data, however, to improve upon this uniform assumption, the distribution of haematocrit (after diffusion/transport/shear effects) at the outlet is copied periodically every 0.1 s to be the inlet condition in an attempt to reach a more physiological distribution. The final distribution at the outlet is available in electronic supplementary material, figure S1.

### 3.3. Haemodynamic parameters

To assess the impact of surface roughness, and evaluate the effects of viscosity on the near-wall haemodynamics the following parameters were defined, as follows:

3.12 $$\tau_{w}=\mu {\frac{\partial u_{t}}{\partial n}|}_{\mathrm{wall}},$$

where *τ_w_* is WSS, *u_t_* is the tangential wall velocity and *n* is the unit vector perpendicular to the wall.

3.13 $${\bar{\tau }}_{w}=\frac{1}{T}\int_{0}^{T}|\tau_{w}|\;\;\text{d}t,$$

where ${\bar{\tau }}_{w}\;$is the time-averaged WSS (TAWSS) over the length of the cardiac cycle, *T*

3.14 $${\bar{\tau }}_{aaw}=\frac{1}{A}\overset{n}{\underset{i=1}{\sum }}{\bar{\tau }}_{w}|A_{i}|,$$

where ${\bar{\tau }}_{aaw}\;$is the area-averaged TAWSS (AATAWSS) which provides an overall magnitude for each rough/smooth segment defined previously.

3.15 $$\theta_{i}=\frac{1}{2}\left(1-\frac{\left|\int_{0}^{T}\tau_{w}\;\text{d}t\right|}{\int_{0}^{T}|\tau_{w}|\;\text{d}t}\right),$$

where *θ_i_* is the oscillatory shear index (OSI), a dimensionless parameter introduced by He & Ku [73] where values close to 0.5 indicate flow oscillation, and values near 0 indicate no flow reversal.

3.16 $$t_{r}=\frac{k}{(1-2\theta_{i}){\bar{\tau }}_{w}},$$

where *t_r_* is the relative residence time (RRT), which provides a measure of the time fluid spends in an arbitrary near-wall region first introduced by Himburg *et al.* [74]. The proportionality constant *k* arises from the near-wall assumption and is set as *k* = 1.

3.17 $$I_{L}=\frac{\mu }{\mu_{N}},$$

where *I_L_* is the local non-Newtonian importance factor (NNIF) first introduced by Ballyk *et al.* [52] where values outside of unity indicate the presence of non-Newtonian effects. The Newtonian viscosity of blood is set as that of the Newtonian model in table 1 as *μ_N_* = 3.45 mPa.s.

The two multiphase models (Quemada–Das and MKM5) allow for the interactions between constituent parts of blood and due to their separate material definitions, each phase has its own associated variables such as velocity, shear, viscosity and volume fraction. To calculate the same haemodynamic parameters above (equations (3.12)–(3.17)) for the multiphase models, the volume fraction of each phase is considered so the properties in a given cell are weighted between the two phases as given by equation (3.18).

3.18 $$A_{m}=\emptyset_{rbc}A_{rbc}+\emptyset_{\mathrm{plasma}}A_{\mathrm{plasma}}\;,$$

where *A* denotes any physical property of the phase/mixture.

### 3.4. Solver settings

The governing equations were solved iteratively using the discrete form of the SIMPLE algorithm for the pressure–velocity coupling (phase-coupled for the multiphase models [75]), with a first-order time discretization. Computation was performed as a distributed process across multiple cores on high-performance computing (HPC) nodes, with optimized efficiency through a customized message passing interface (MPI). Solution parameters and machine specifications for the solutions are listed in table 2. A smaller time step was chosen for the multiphase models to avoid initial instabilities due to the nonlinearity of the RBC viscosity models. The models were solved for a total simulation time of 5.6 s corresponding to seven cardiac cycles, with the multiphase model chosen to have a smaller time step for improved phase interactions.

**Table 2.** Convergence parameters and solution information for the single and multiphase models.

phase	cardiac cycles	time step (s)	minimum continuity residual	HPC machines	clock time (hours)
single	7	0.005	10^−5^	300 Intel Xeon ES-2640 v4 processors with 2TB DDR4 RAM	24
multi		0.001			300

## 4. Results

### 4.1. Single-phase

The haemodynamic parameters defined above (equations (3.12)–(3.17)) were evaluated across the seventh cardiac cycle, on the opposing rough/smooth segments of the artery (figure 1) and shown in table 3.

**Table 3.** Haemodynamic parameters on the rough (R) and smooth (S) segments of the artery for all single-phase models.

parameter	Newtonian				Carreau–Yasuda		generalized power law	
	*R*	*S*	*R*	*S*	*R*	*S*	*R*	*S*
AATAWSS (pa)	1.30	1.31	1.41	1.42	1.42	1.44	1.34	1.34
max WSS (pa)	6.50	2.68	5.58	3.40	6.29	3.26	6.51	3.00
min WSS (pa)	0.027	0.460	0.052	0.168	0.060	0.391	0.059	0.409
max RRT (pa^−1^)	34.4	0.7	12.0	0.8	14.4	0.7	15.5	0.7
max OSI	0.012	3.4 × 10^−5^	0.012	4.8 × 10^−5^	0.012	3.7 × 10^−5^	0.012	3.6 × 10^−5^
max NNIF	1	1	2.5	1.4	2	1.3	2.8	1.3
max shear (s^−1^)	1826	773	1504	925	1718	899	1800	851
min shear (s^−1^)	9.69	125.89	7.52	36.97	6.85	90.62	7.13	92.84

The AATAWSS for the rough/smooth segments differs by less than 1% in each single-phase model, despite the much wider range of instantaneous WSS for the rough segment. Given the two segments lie on the same artery, the average magnitudes of WSS are expected to be similar. The two Carreau type models have the most similar predictions of WSS, with the generalized power law more closely agreeing with the Newtonian model. All single-phase models predict the same distribution of haemodynamic parameters in table 3, with only the magnitudes and ranges differing between each model.

**Figure 6.**
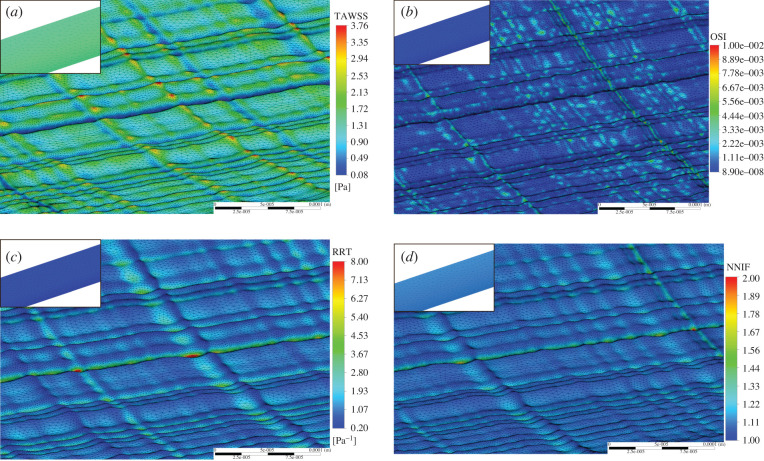
Contours of (*a*) time averaged wall shear stress (TAWSS—generalized power law), (*b*) oscillatory shear index (OSI—Carreau), (*c*) relative residence time (RRT—Newtonian) and (*d*) non-Newtonian importance factor (NNIF—Carreau–Yasuda) for the rough surfaced coronary artery segments with the distributions for the corresponding smooth segments inlaid as reference.

The TAWSS on the rough segment varies by more than double that of the smooth, with ‘peaks’ and ‘troughs’ of the roughness experiencing a TAWSS of around 3.7 Pa and 0.08 Pa, respectively, for all single-phase models, with the distribution for the generalized power law shown in figure 6*a*.

Both the maximum OSI and RRT are significantly greater over the rough surface than the smooth surface, with the largest RRT occurring in the Newtonian model being more than double the closest non-Newtonian model. Overall, the magnitude of the OSI is negligible which is to be expected given the straight cylindrical nature of this artery segment, with a uniform distribution on the smooth segment and a seemingly random pattern of OSI on the rough segment, with concentrations around peaks in the circumferential roughness shown for the Carreau model in figure 6*b*. The RRT distribution is uniform over the smooth segment in all single-phase models, with a magnitude ≈10× smaller than the rough segment. Maximum RRT occurs unsurprisingly in the ‘troughs’ of the roughness as the velocity in these areas approaches zero, coupled with the lowest shear rates of between 7–10 s^−1^ resulting in an increased viscosity and minimal blood washout, with the distribution shown for the Newtonian model shown in figure 6*c*.

The non-Newtonian effects are much more pronounced on the rough arterial segments, with the generalized power law model predicting a peak $I_{L}=2.8$ despite the lack of complex geometry features such as plaques, bifurcations or curvature. This range of NNIF is explained by the wide range of shear rates occurring over the rough segment. The largest range of shear rates on the rough and smooth surfaces occurs with the Newtonian and Carreau models respectively; however, the generalized power law has the greatest combined range of shear rates, and thus the greatest NNIF value. The distribution of NNIF is uniform across the smooth segment, with a similar magnitude over the majority of the rough surface, except for the troughs of the roughness where the NNIF reaches its maximum due to the low shear conditions. The NNIF distribution is shown for the Carreau–Yasuda model in figure 6*d*.

### 4.2. Multiphase

All parameters in table 4 are calculated using the mixture relationship (equation 3.18) for best comparisons between the single and multiphase models, with maximum/minimum values reported across the final cardiac cycle.

**Table 4.** Haemodynamic and multiphase parameters for the rough (R) and smooth (S) segments for both multiphase models.

parameter	Quemada–Das		modified Krieger model 5 parameters (MKM5)	
	*R*	*S*	*R*	*S*
AATAWSS (Pa)	1.048	0.918	1.481	1.317
max WSS (Pa)	243.6	1.57	157	1.85
min WSS (Pa)	0.018	0.515	0.029	0.478
max RRT (Pa^−1^)	72.9	1.1	14.6	0.8
max OSI	3.6 × 10^−1^	7.75 × 10^−7^	2.25 × 10^−1^	1.01 × 10^−6^
max NNIF	718.6	1.48	2.14 × 10^5^	2.22
max shear (s^−1^)	3.65 × 10^4^	721	3.58 × 10^4^	862
min shear (s^−1^)	0.22	213	2.17	138
max haematocrit	0.968	0.467	0.980	0.469
time-averaged haematocrit	0.461	0.459	0.456	0.461
time-averaged mixture viscosity (Pa.s)	0.005184		0.007515	

**Figure 7.**
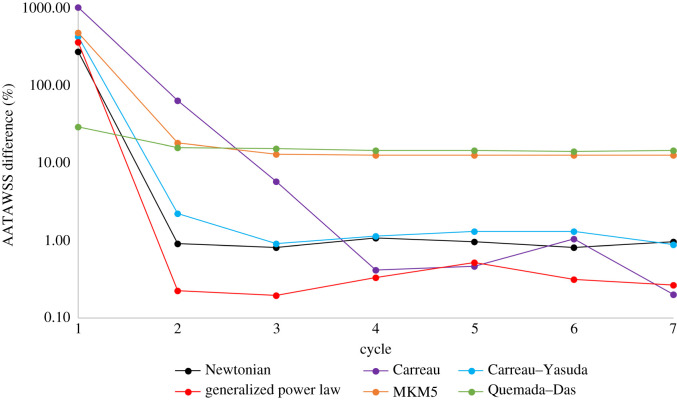
Difference in AATAWSS for the rough/smooth segments for each model.

To compare the difference in AATAWSS on the rough/smooth segments the relative difference between the final magnitudes at the end of each cardiac cycle was calculated using equation (4.1) and plotted below in figure 7.

4.1 $${\bar{\tau }}_{aaw,\mathrm{diff}}=\frac{\left|{\bar{\tau }}_{aaw,r}-{\bar{\tau }}_{aaw,s}\right|}{\left|{\bar{\tau }}_{aaw,s}\right|}.$$

**Figure 8.**
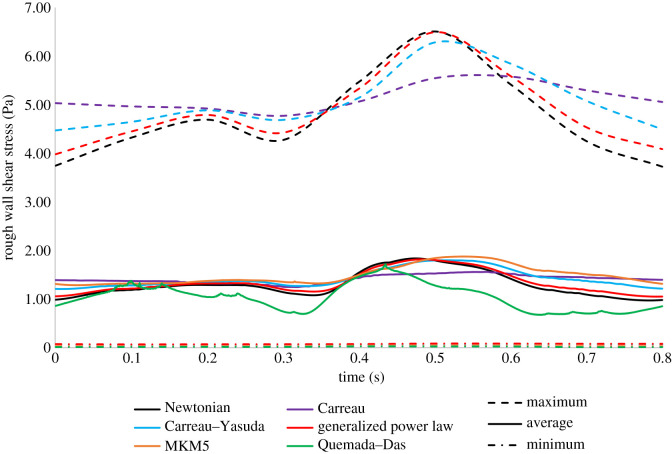
Variation of rough wall shear stress (WSS) with time, maximum, average and minimum values of WSS on the rough segment for each model are presented. (Max values for multiphase models not included due to magnitude.)

**Figure 9.**
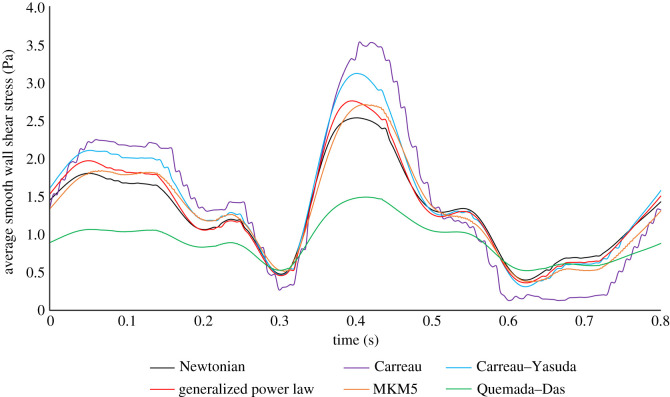
Variation of wall shear stress with time averaged over the smooth segment for each model are presented. (Max and Min values not reported because of uniform nature of stress distribution.)

The difference in AATAWSS between the rough/smooth segments for both multiphase models is around 13%, compared to the less than 1% in the single-phase models. The MKM5 model's prediction of WSS parameters is most similar to the single-phase models, however, the inclusion of haematocrit in viscous calculations greatly increases the maximum WSS and NNIF for both multiphase models. The AATAWSS magnitudes for the Quemada–Das model are the lowest in this study for both the rough/smooth segments, despite a stress distribution similar to the generalized power law model in figure 6*a*. The variation of WSS for the rough/smooth segments for all models is plotted in figures 8 and 9. For the smooth segment, the variation in WSS is clearly proportional to the velocity of the flow, and for every model, the WSS follows this trend shown in figure 5. The rough segment shows less of a change in WSS during the cycle, with a much more consistent average, maximum and minimum WSS over the surface, with the maxima occurring around the time of peak velocity.

The magnitudes of RRT and OSI are greater in both multiphase models for both the rough/smooth segments, with maximum OSI values approaching the 0.5 limit indicating regions of highly oscillatory shear stress. The wide variation in haematocrit shown over the surface in figure 11 results in two extremes of viscous prediction and thus the extreme values of NNIF. Despite these extreme variations in local shear of the RBC's and haematocrit over the rough surface, the overall average magnitudes of AATAWSS, RRT, haematocrit and mixture viscosity remain unaffected, implying that these extremes are localized to very small regions.

**Figure 10.**
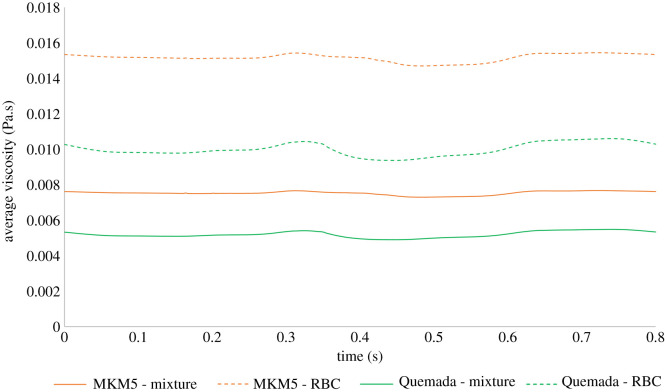
Variation of average viscosity with time. Variation of mixture and RBC viscosity during the cardiac cycle are presented.

The overall mixture viscosity predicted by the Quemada–Das model was around 30% lower than that of the MKM5 model which is expected given the shear graphs plotted for the inlet haematocrit in figure 4, with the variation during the cardiac cycle shown in figure 10.

**Figure 11.**
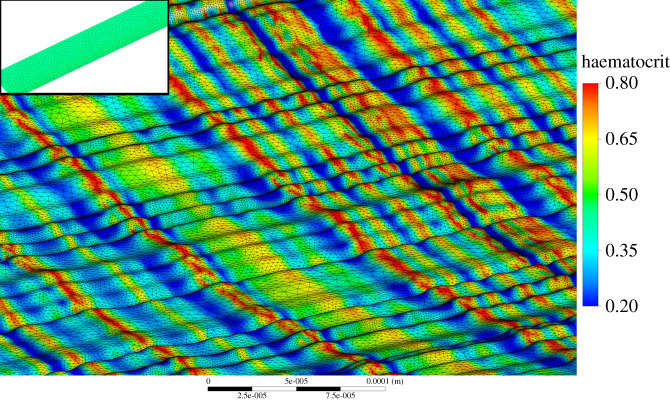
Haematocrit distribution over the rough segment (smooth inlaid) for the MKM5 model.

**Figure 12.**
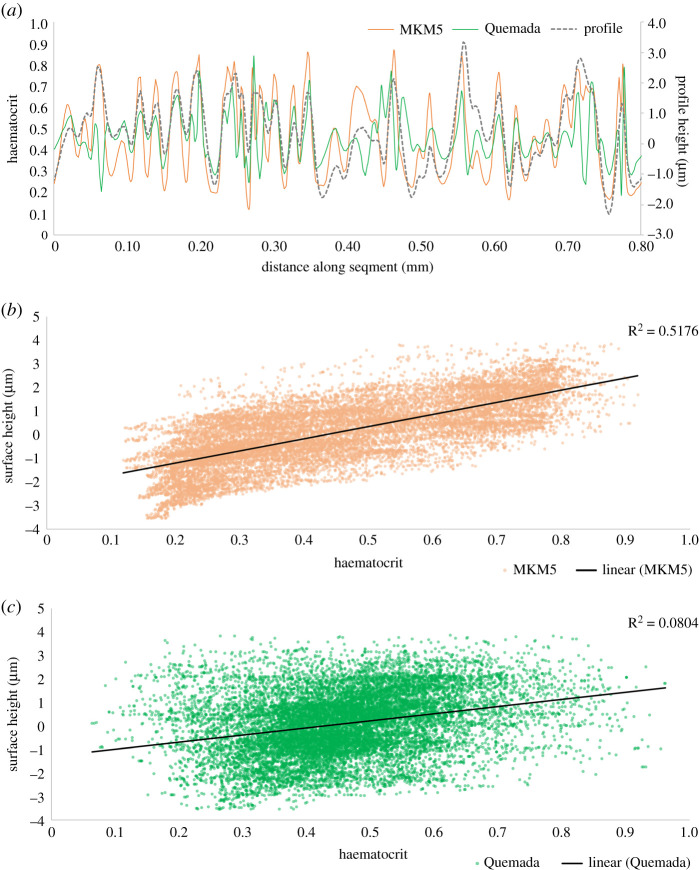
(*a*) Variation of surface haematocrit and height along a rough segment. (Data taken from five lines over the surface, parallel to flow direction.) (*b*,*c*) Scatter plots of surface height versus haematocrit for the MKM5 and Quemada models, respectively.

The haematocrit distribution over the smooth segment is uniform around 0.46 in both models, however, the rough segment varies significantly with peaks in haematocrit occurring with peaks in the roughness (figures 11 and 12) similar to the OSI and TAWSS distribution of the single-phase models. Areas with extremes of haematocrit will also experience either high or low viscosity (figure 4) which will in turn affect haemodynamic parameters such as NNIF and WSS. Variations in haematocrit over the rough surface after 4.8 s are further detailed in Figures 11 and 12.

The impact of surface roughness on the haematocrit is clearly seen in figure 12*a*, where large changes in surface height (e.g. −1 µm to 3 µm) result in a significant increase in haematocrit for both multiphase models (0.2–0.8).

## 5. Discussion

Simulations of blood flow in a coronary artery with a partially rough surface have been performed to investigate the effect of a realistic surface texture on conventional haemodynamic parameters. Overall, the rough surface had a greater variation in the parameters reported in tables 3 and 4 in particular the WSS, RRT and shear rate, with values averaged across the entire rough/smooth segments being similar. The smooth-walled assumption has been previously used in all cardiovascular models to date, which results in a uniform distribution of the haemodynamic parameters shown in figure 6 compared to the variation seen for all parameters on the rough segment. As WSS is closely linked to the onset of atherosclerosis, an increase in lumen roughness will lead to larger regions with low WSS which will then become more susceptible to the onset of atherosclerotic lesions.

### 5.1. Rough surface effects

Near-wall haemodynamics are known to relate to the onset and progression of CVDs [76], with computational approaches typically focusing on Lagrangian particle tracking methods within this region [77,78], or Eulerian models evaluating the WSS/advection/diffusion close to the wall [76,79], yet these do not include the endothelial surface texture. This simplification arises because the region of interest for coronary models is typically macroscopic, focussing on the effects of stenosis or bifurcations where the surface texture can be ignored.

For a straight cylindrical section of the coronary artery, the physiological WSS magnitude varies between [6] 1 and 7 Pa with a TAWSS magnitude of approximately [1,80] 1.5 Pa. According to a clinical *in vivo* study of 506 patients, an AATAWSS magnitude of 1–1.7 Pa can be considered moderate [81], with all models predicting values between 1.3 and1.48 Pa for both the rough/smooth segments except for the Quemada which was around 1 Pa. For further validation, an estimate of WSS assuming Poiseuille flow for this diameter is 1.39 Pa and therefore all single-phase models provide a physiological magnitude of both WSS and AATAWSS on both the rough/smooth segments.

The surface roughness of the coronary artery has been examined as a factor in the onset of atherosclerosis [23–25], where the lumen roughness increases due to endothelial damage [26], and so models that focus on predicting likely sites where atherosclerosis may form [82,83], or modelling the accumulation of lipids/monocytes [84] in the artery may benefit from considering this locally in those regions. In addition, an *in vitro* study using human cultured endothelium of the carotid artery showed increased particulate adhesion in the presence of oscillating shear stress [85]. This is similar to the findings in this study (e.g. figures 6*b*, 11 and 12*a*), where regions displaying peak OSI values appear to correspond to the highest haematocrit as RBCs accumulate. Furthermore, the elevated WSS at these peaks would impact on other blood particulates such as platelets, where increased WSS and shear forces are pivotal in the activation and adhesion of platelets [86].

While full-scale arterial models with this texture are still computationally unfeasible, the technique could be applied to local regions to study interactions with medical devices such as stents [87,88] or haemodialysis catheters [89,90]. With coronary artery stents having a thickness of approximately [59] 100 µm, the variation in surface roughness for a diseased artery may impact on the local haemodynamics around the struts.

While parameters averaged over the entire segments are comparable, the much wider range occurring over the undulations in the rough surface result in a maximum/minimum WSS of around 6.4 Pa and 0.05 Pa for all the rheological models. With regions of higher WSS thought to be atheroprotective [91,92], the AATAWSS for both segments is sufficiently high to indicate a healthy functioning artery [6], however, the ‘troughs’ of the roughness indicate regions with a low enough WSS where endothelial dysfunction may be a consideration even in this idealized geometry. These effects would likely be exacerbated by arterial curvature/stenosis, especially as the surface roughness increases when the endothelium is damaged [26].

### 5.2. Rheological model effects

As the surface texture has yet to be modelled using CFD, a comparison of the most common approaches to blood rheology has also been performed, to assess their suitability for such multi-scale models. Since blood exhibits both Newtonian and non-Newtonian properties, the choice of rheological model describing this behaviour can greatly impact on the assessment of haemodynamic parameters [51,59,84], with the majority of models being single-phase, whose viscosity only varies under shear, with fewer models considering phase interactions between plasma/RBCs and the effects of haematocrit. Previous comparisons between models for coronary arteries have shown that a Newtonian assumption consistently underestimates the magnitude of WSS [51,93], and with the introduction of a non-Newtonian importance factor by Ballyk *et al.* [52], it has been shown that specific areas of the coronary artery (curvature/stenosis/bifurcations) experience significant regions of non-Newtonian flow [58,60,94] and as such an appropriate model should be selected. Even with multiple comparisons between viscous models [50,95–97], it is still challenging to determine which non-Newtonian model is most suitable for coronary models, and indeed likely depends on the focus of the study and the pathology in question.

All single-phase models show a large range of shear rates occurring over both the rough/smooth segments (table 3), with the NNIF indicating again that even under normal physiological conditions blood exhibits significant non-Newtonian properties. At higher shear rates (greater than 500 s^−1^) all single-phase models predict a similar viscosity of around 3.45 mPa.s, yet it is at the lower shear rates (less than 100 s^−1^) where predictions of viscosity diverge (figure 4) impacting the NNIF as well as the conventional parameters used to assess atherosclerotic/stenotic regions. This low shear behaviour is crucial to modelling the rough surface accurately [48,51], yet physiologically accurate viscometer measurements for blood at low shear are difficult due to inherent inaccuracies as well as blood's dependence on temperature and haematocrit [45,51].

While the single-phase models might oversimplify the near-wall/low-shear haemodynamics occurring on the rough surface, a multiphase model which considers haematocrit and local RBC transport may improve upon this. The MKM5 multiphase model performs most similarly to the single-phase models, with all parameters for both the rough/smooth segments being in close agreement apart from Max WSS/OSI/Shear/NNIF on the rough segment. This is likely due to the instability of the multiphase models at extremes of haematocrit [54]. Above a certain haematocrit, blood ceases to behave as a fluid and the accumulation of RBCs alters the local haemodynamics in these regions [37]. This aggregation process is dependent on a range of biochemical factors [54,98], and is not accounted for in the MKM5 model resulting in predictions of high haematocrit/shear (figures 11 and 12*a*) and hence much higher WSS/OSI/NNIF values in these regions. While the link between surface roughness and haematocrit is clearly shown in figure 12*a*, the large scatter distribution in Figure 12*b*,*c* indicates the influence of additional parameters in this near-wall region which might include: low shear rate, flow stagnation and localized regions of high/low viscosity. As plasma is less viscous than RBCs, it exerts a lower WSS magnitude, hence, the presence of an RBC depleted region will promote poor endothelial function. As individual RBCs are not simulated with this approach, additional effects due to the elastic deformation of RBCs [99,100] may further modify these micro-scale near-wall haemodynamics as RBC's deform around the surface texture. While this model combines the variation in lumen roughness with multiphase cellular transport, to further link the two scales, additional constraints on near-wall flow such as advection/diffusion at the endothelial surface or the aggregation/deformation of RBCs would be desirable to better understand how RBC transport effects these shear based parameters.

While all measures have been taken to ensure the accuracy of the presented model, the study has certain limitations/assumptions. The main limitation of the study was the use of a simple cylindrical shape, while based upon *in vivo* dimensions, the lack of any features such as arterial curvature/stenosis, bifurcations or methods to induce flow disturbance will compromise the development of natural recirculation/oscillations. While this is detrimental to the physiological accuracy, the focus was the impact of realistic surface roughness, and this geometry enabled controlled comparisons to be taken between the two surface types. As the use of surface roughness in a 3D model has now been established, similar methodologies could be applied to more clinically relevant applications, such as increasing surface roughness as a precursor to arterial dysfunction, particle migration in the low shear environments downstream of bifurcations and interactions between stents and a rough-surfaced artery. Computation of maximum WSS values was performed pointwise, and while this provides sensible results for all single-phase models, the resulting maximum WSS values for the multiphase models were higher than expected for a physiological model. This arose due to extremes of haematocrit occurring in the near-wall region, in part potentially due to a lack of physical laws governing aggregation of RBCs. One approach to reducing the multiphase WSS predictions would have been to average these values over a wider patch. However, for consistency between all models (and to avoid introducing an artefact to non-multiphase models), such a method was not used; additionally, such an approach would require the use of a parameter (e.g. patch area), the value of which would need to be arbitrarily chosen.

## 6. Conclusion

Comparisons of well-established haemodynamic parameters used in coronary artery models on both a smooth and realistically rough artery surface have shown that a rough surface results in a greater range of values, and averages over the rough/smooth segments are in good agreement. The combination of macro-micro scales to evaluate coronary flow over these rough surfaces has highlighted how complex the near-wall haemodynamics can be, even in a geometrically and physiologically simple case. The different approaches to rheology applied to this surface show that the single-phase models provide a stable estimate of local haemodynamics as seen in other studies, yet oversimplify the complex behaviour occurring over the undulations at the lumen surface, particularly in the Newtonian case. The use of multiphase models attempts to further characterize the behaviour of blood in the low-shear/micro-scale roughness region, and while the MKM5 appears more suitable than the Quemada–Das, to truly encompass the behaviour of blood/particulates at this boundary requires considering additional phenomena such as advection/diffusion at the endothelium, RBC aggregation/deformation and the effects of fibrogen/plasma protein concentrations.

## Supplementary Material

Cross section of hematocrit at outlet

## Supplementary Material

CAD File of rough segment

## Data accessibility

All relevant data are within the manuscript and its supporting files.
