# Supplementary figures and images for: Assessment of surface roughness and blood rheology on local coronary haemodynamics: a multi-scale computational fluid dynamics study
Source: J R Soc Interface. 2020 Aug 12;17(169):20200327. doi: 10.1098/rsif.2020.0327 (PMC7482556; doi:10.1098/rsif.2020.0327)

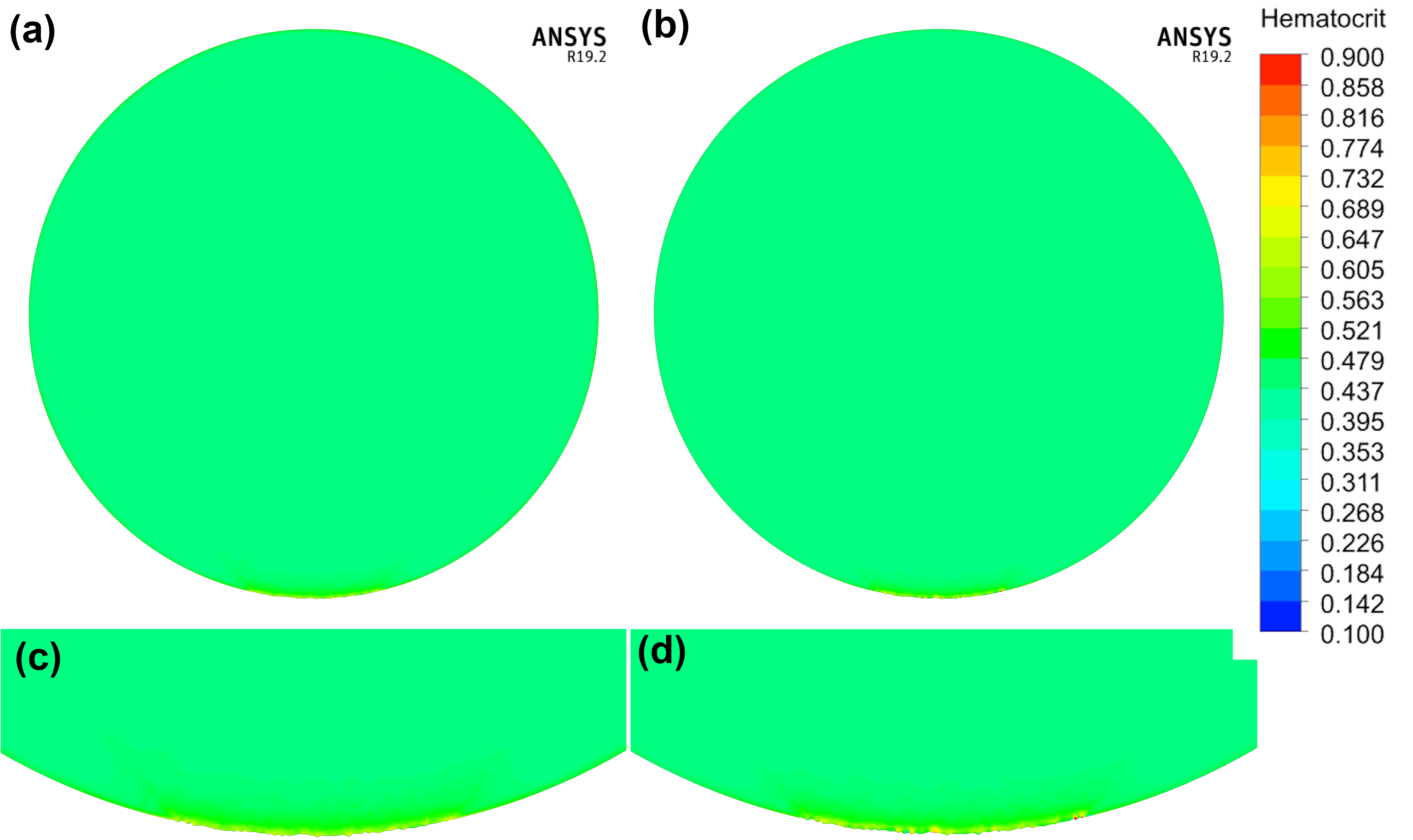

Supplement: Cross section of hematocrit at outlet [file rsif20200327supp1.jpg]
